# Reduced Apaf-1 expression in human cutaneous melanomas

**DOI:** 10.1038/sj.bjc.6602092

**Published:** 2004-08-10

**Authors:** D L Dai, M Martinka, J A Bush, G Li

**Affiliations:** 1 Department of Medicine, Division of Dermatology, Vancouver Hospital and Health Sciences Centre, University of British Columbia, Vancouver, BC, Canada V6H 3Z6; 2 Department of Pathology, Vancouver Hospital and Health Sciences Centre, University of British Columbia, Vancouver, BC, Canada V6H 3Z6

**Keywords:** Apaf-1, tissue microarray, melanoma, gene expression

## Abstract

Malignant melanoma is a life-threatening skin cancer due to its highly metastatic character and resistance to radio- and chemotherapy. It is believed that the ability to evade apoptosis is the key mechanism for the rapid growth of cancer cells. However, the exact mechanism for failure in the apoptotic pathway in melanoma cells is unclear. *p53*, the most frequently mutated tumour suppressor gene in human cancers, is a key apoptosis inducer. However, *p53* mutation is only found in 15–20% of melanoma biopsies. Recently, it was found that Apaf-1, a downstream target of p53, is inactivated in metastatic melanoma. Specifically, loss of heterozygosity (LOH) of the Apaf-1 gene was found in 40% of metastatic melanoma. To determine if loss of Apaf-1 expression is indeed involved in melanoma progression, we employed the tissue microarray technology and examined Apaf-1 expression in 70 human primary malignant melanoma biopsies by immunohistochemistry. Our data showed that Apaf-1 expression is significantly reduced in melanoma cells compared with normal nevi (*χ*^2^=6.02, *P*=0.014). Our results also revealed that loss of Apaf-1 was not associated with the tumour thickness, ulceration or subtype, patient's gender, age and 5-year survival. In addition, our *in vitro* apoptosis assay revealed that overexpression of Apaf-1 can sensitise melanoma cells to anticancer drug treatment. Taken together, our data indicate that Apaf-1 expression is significantly reduced in human melanoma and that Apaf-1 may serve as a therapeutic target in melanoma.

Cutaneous malignant melanoma is a deadly skin cancer, which is derived from epidermal melanocytes. The incidence of melanoma is increasing more rapidly than any other tumours among Caucasian populations (Rigel *et al*, 1996). It is estimated that the lifetime risk for melanoma has increased by 15-fold in the past 60 years, reaching one in 68 in America (Glass and Hoover, 1989; Koh and Geller, 1995; Jemal *et al*, 2002, Rigel, 2002). Although early melanomas are curable with surgical excision (Balch *et al*, 2001), up to 20% of patients will develop metastatic tumours due to its high capability of invasion and rapid metastasis to other organs (Houghton and Polsky, 2002). Patients with metastatic melanoma have a poor prognosis, with a median survival of only 6–10 months (Jemal *et al*, 2002).

The main obstacle in treating melanoma is its resistant characteristic to conventional chemotherapy, with an overall response rate of only less than 20% (Gilchrest *et al*, 1999; Li and McClay, 2002). Although the molecular mechanism for drug resistance in melanoma is still poorly understood, it appears that the low therapeutic efficacy in this disease likely relates to a relative inability to induce apoptosis (Li *et al*, 1998; Soengas and Lowe, 2003). In addition, resistance to apoptosis has been correlated with increased metastatic potential in melanoma (Glinsky *et al*, 1997). p53, a central sensor linking DNA damage to apoptosis, has been related to tumorigenesis and chemoresistance in many tumour types (Fridman and Lowe, 2003; Oren, 2003), including melanoma (Li *et al*, 2000). However, melanomas display a very low rate of p53 mutations (Albino *et al*, 1994; Montano *et al*, 1994; Ragnarsson-Olding *et al*, 2002) despite their extreme chemoresistance. Thus, dysregulation of other components of the p53-mediated pathway in melanoma may also contribute to disruption of apoptosis. In fact, altered expression of downstream effectors of p53 such as IAP family members (Grossman *et al*, 1999) and Bcl-2 family members (Bush and Li, 2003; Hakansson *et al*, 2003) have been reported in melanoma. Recently, Apaf-1 gene, a downstream effector of p53, which links release of cytochrome *c* to activation of caspase-9 in mitochondrion-mediated apoptosis pathway (Li *et al*, 1997), was found to be inactivated in melanoma presumably by methylation (Soengas *et al*, 2001). Specifically, LOH of Apaf-1 alleles was detected in 42% of the metastatic melanoma specimens and more than 50% of cell lines that derived from metastatic melanoma showed negative Apaf-1 expression. Strikingly, restoring physiological levels of Apaf-1 through gene transfer or treatment with methylation inhibitor can dramatically enhances chemosensitivity in Apaf-1-deficient cell lines (Soengas *et al*, 2001), which raises the possibility that restoring Apaf-1 regulation to some melanomas would have therapeutic benefit.

To further investigate the role of Apaf-1 in melanoma progression, we, used tissue microarray (TMA) technology and immunohistochemistry in the present study, to evaluate the Apaf-1 expression level in primary human melanoma at different stages. Our data showed that Apaf-1 expression is significantly reduced in primary human melanomas compared to normal nevi (*P*=0.014). However, reduced Apaf-1 expression is not correlated with melanoma thickness or 5-year patient survival. Also, our *in vitro* cell survival and apoptosis assays demonstrated that overexpression of Apaf-1 in melanoma cells enhanced anticancer drug-induced apoptosis, suggesting that Apaf-1 may serve as a therapeutic target in melanoma.

## MATERIAL AND METHODS

### TMA construction

Formalin-fixed, paraffin-embedded tissues from 87 human primary melanomas and 16 nevi were used for our present study. All specimens were obtained from the 1990 to 1997 archives of the Department of Pathology, Vancouver General Hospital. The most representative tumour area was carefully selected and marked on the H&E-stained slide. The TMAs were assembled using a tissue-array instrument (Beecher Instruments, Silver Spring, MD, USA) consisting of thin-walled stainless steel punches and stylets used to empty and transfer the needle content. The assembly was held in an *X*–*Y* position guide equipped with semiautomatic micrometers, with a 1-mm increment between individual samples and 3-mm punch depth stop device. Briefly, the instrument was used to create holes in a recipient block with defined array cores. A solid stylet, which closely fits the needle, was used to transfer the tissue cores into the recipient block. Taking into account the limitations of the representative areas of the tumour, we used duplicate or triplicate 0.6-mm diameter tissue cores from each donor block. Multiple 4-*μ*m sections were cut with a Leica microtome. Sections were transferred to adhesive-coated slides using routine histology procedures. One section was routinely deparaffinised with standard xylene and hydrated through graded ethanol in water, then stained with H&E and covered with a coverslip. The remaining sections were stored at room temperature for immunohistochemistry staining.

### Immunohistochemistry of TMA

The TMA slides were dewaxed by heating at 55°C for 30 min and by three washes, 5 min each, with xylene. Tissues were rehydrated by a series of 5-min washes in 100, 90 and 70% ethanol and phosphate-buffered saline (PBS). Antigen retrieval was performed by microwaving the samples for 4 min, 20 s at full power in 250 ml of 10 mM sodium citrate (pH 6.0). Endogenous peroxidase activity was blocked with 0.3% hydrogen peroxide for 20 min. Nonspecific binding was blocked with goat serum for 30 min. The primary polyclonal rabbit anti-Apaf-1 antibody (BD Biosciences, Mississauga, Ontario, Canada) was diluted 1 : 300 using goat serum and incubated at room temperature for 1 h. After three washes, 2 min each with PBS, the sections were incubated with a biotinylated goat anti-rabbit secondary antibody for 30 min (Santa Cruz Biotechnology, Santa Cruz, CA, USA). After three washes, 2 min each with PBS, horseradish peroxidase-streptavidin (Santa Cruz Biotechnology) was added to the section for 30 min, followed by another three washes, 2 min each with PBS. The samples were developed with 3,3′-diaminobenzidine substrate (Vector Laboratories, Burlington, Ontario, Canada) for 7 min and counterstained with haematoxylin. Then, the slides were dehydrated following a standard procedure and sealed with coverslips. Negative controls were performed by omitting Apaf-1 antibody during the primary antibody incubation.

### Evaluation of immunostaining

The Apaf-1 staining in TMA was examined blinded by three independent observers (including one dermatopathologist) simultaneously, and a consensus score was reached for each core. Due to loss of biopsy cores, 70 cases of primary melanoma and 13 cases of nevi could be evaluated for Apaf-1 staining. The staining intensity was scored as negative (0), weak staining (1+), moderate staining (2+) and strong staining (3+). There was a high level of consistency of immunohistochemical staining between the duplicate or triplicate cores in the TMAs. In total, 78% of the biopsies had uniform staining between different cores. For the other 22% of cases that had one level of difference in staining between cores, the higher score was used for statistical analysis. The reason for differential staining in some biopsies could be that melanoma is a heterogeneous tumour, so different areas in the tumour may represent different stages of tumour progression.

### Statistical analysis of TMA

Statistical analysis was performed with the SPSS 11.5 software (SPSS, Chicago, IL, USA). The *χ*^2^-test was used to compare the quantitative differences of Apaf-1 expression in tumours and nevi. The association between the Apaf-1 expression and the clinicopathological parameters, including age, sex, tumour thickness, location, histological subtype and tumour ulceration, was also evaluated by *χ*^2^ test. The relationship between Apaf-1 expression and 5-year survival was assessed by log-rank test. A *P*-value of <0.05 was considered significant.

### Cell culture and transfection

Melanoma cell lines MMRU (p53^WT^) and MEWO (p53^MUT^) were grown at 37°C in Dulbecco's modified Eagle's medium containing 10% fetal bovine serum in an atmosphere containing 5% CO_2._ For transfection, cells were grown to 50–60% confluency. The ratio of 1 *μ*g DNA : 25 *μ*l Effectene reagent (Qiagen, Mississauga, Ontario, Canada) was used for transfection. Plasmids used for transfection included pEGFP-N1 control plasmid (BD Clontech, Palo Alto, CA, USA) and pcDNA3-Apaf-1 (a kind gift from Dr K Bhalla, H Lee Moffitt Cancer Centre, Tampa, FL, USA).

### Western blot analysis

Cells were washed with PBS three times, and lysed in triple-detergent buffer (50 mM Tris-Cl (pH 8.0), 150 mM NaCl, 0.02% sodium azide, 0.1% SDS, 100 *μ*g ml^−1^ phenylmethylsulphonyl fluoride, 1 *μ*g ml^−1^ aprotinin, 1% Nonidet P-40, 0.5% sodium deoxycholate) for 20 min on ice. The lysate was centrifuged at 12 000 *g* for 10 min and the supernatant was collected. The protein concentration was determined by the DC Protein Assay (Bio-Rad, Mississauga, Ontario, Canada). In total, 50 *μ*g lane^−1^ of proteins were separated on 6.5% polyacrylamide/SDS gels and electroblotted onto polyvinylidene difluoride filters. The filters were then blocked with 5% skimmed milk for 1 h and incubated with 1 : 1000 polyclonal rabbit anti-Apaf-1 antibody (BD Biosciences, Mississauga, Ontario, Canada) for 1 h at room temperature, and then incubated with horseradish peroxidase-conjugated secondary antibody for 1 h at room temperature. The signals were detected with SuperSignal enhanced chemiluminescence (Pierce, Rockford, IL, USA).

### Cell survival assay

MMRU cells were grown to 50–60% confluency in 24-well plates and transfected with pEGFP-N1 or pcDNA3-Apaf-1 plasmid. At 24 h after transfection, the cells were treated with 500 nM doxorubicin (DOX), 800 nM vincristine (VIN) or 200 nM camptothecin (CPT) (Sigma, Mississauga, Ontario, Canada) for another 24 h and the cell survival was determined by sulphorhodamine (SRB) assay (Bush *et al*, 2001). Briefly, the medium was removed and the cells were fixed with 10% trichloroacetate for 1 h at 4°C, rinsed five times with water, and air-dried. The cells were then fixed with 0.4% suphforhodamine B (SRB) in 1% acetic acid for 20 min. After rinsing four times with 1% acetic acid and air dried, 500 *μ*l of 10 mM Tris (pH 10.5) was added to the wells for 30 min. The colorimetric reading was carried out in a Microplate Autoreader (Bio-Tek Instruments Inc., Winooski, VT, USA) at 550 nm.

### Enzyme-linked immunosorbent assay (ELISA) of apoptosis

MMRU and MEWO cells were grown to 50–60% confluency in 24-well plates and transfected with pEGFP-N1 or pcDNA3-Apaf-1 plasmid. At 24 h after transfection, the cells were treated with or without 200 nM CPT for 24 h. The ELISA was then performed using a Cell Death Detection Elisa^plus^ kit (Boehringer Mannheim, Quebec, Canada) according to the manufacturer's protocol. The colorimetric analysis was carried out in a Microplate Autoreader (Bio-Tek Instruments Inc., Winooski, VT, USA) at 405 nm.

## RESULTS

### Clinicopathological features of TMA

For the 70 primary melanoma cases in which Apaf-1 staining was available, there were 38 male and 32 female, with ages ranging from 21 to 93 years (mean=58). For melanoma staging, we used Breslow thickness as our criteria for evaluating Apaf-1 expression: ⩽0.75 mm, low risk; 0.76–1.5 mm, intermediate risk; 1.51–4.0 mm, high risk; >4.0 mm, very high risk (Marghoob *et al*, 2000). In our study, 14 melanoma cases were ⩽0.75 mm, 39 were 0.76–1.5 mm, 11 were 1.51–4.0 mm, and six were >4.0 mm thick. Among the 70 cases, 32 were superficial spreading melanoma, 12 were lentigo maligna melanoma, and other 26 cases consisted of desmoplastic melanoma, acrolentigous melanoma, and nodular melanoma. In total, 14 melanomas were located in sun-exposed sites (head and neck), and 56 were located in sun-protected sites (trunk, arm, leg, and feet). Tumour ulceration was observed in nine cases (Table 1

**Table 1:** Apaf-1 expression and clinicopathological characteristics of 70 primary melanomas

	**Intensity of Apaf-1 staining**					
	**0**	**1+**	**2+**	**3+**	**Total**	***P***-**value**^a^
*Age*						
⩽57	3 (8%)	20 (56%)	9 (25%)	4 (11%)	36	
>57	3 (9%)	16 (47%)	12 (35%)	3 (9%)	34	*P*>0.05
						
*Sex*						
Male	2 (5%)	18 (47%)	12 (32%)	6 (16%)	38	
Female	4 (13%)	18 (56%)	9 (28%)	1 (3%)	32	*P*>0.05
						
*Tumour thickness (mm)*						
⩽0.75	2 (14%)	6 (43%)	4 (29%)	2 (14%)	14	
0.76–1.5	4 (10%)	20 (51%)	10 (26%)	5 (13%)	39	
1.51–4.0	0 (0%)	7 (64%)	4 (36%)	0 (0%)	11	
>4.0	0 (0%)	3 (50%)	3 (50%)	0 (0%)	6	*P*>0.05^b^
						
*Ulceration*						
Absent	6 (10%)	30 (49%)	18 (30%)	7 (11%)	61	
Present	0 (0%)	6 (67%)	3 (33%)	0 (0%)	9	*P*>0.05
						
*Tumour subtype*^c^						
SSM	4 (13%)	13 (41%)	12 (38%)	3 (9%)	32	
LMM	2 (17%)	5 (42%)	4 (33%)	1 (8%)	12	
Other	0 (0%)	18 (69%)	5 (19%)	3 (12%)	26	*P*>0.05
						
*Site*^d^						
Sun-protected	4 (7%)	28 (50%)	18 (32%)	6 (11%)	56	
Sun-exposed	2 (14%)	8 (57%)	3 (21%)	1 (7%)	14	*P*>0.05

a *χ*^2^ test for low (0, 1+) *vs* high Apaf-1 expression (2+, 3+).

b Tumours ⩽1.5 mm *vs* >1.5 mm.

c SSM=superficial spreading melanoma; LMM=lentigo maligna melanoma; Other includes desmoplastic melanoma, acrolentigous melanoma and nodular melanoma.

d Sun-protected sites: trunk, arm, leg and feet. Sun-exposed sites: head and neck.

).

### Apaf-1 expression in human melanoma

**Figure 1:**
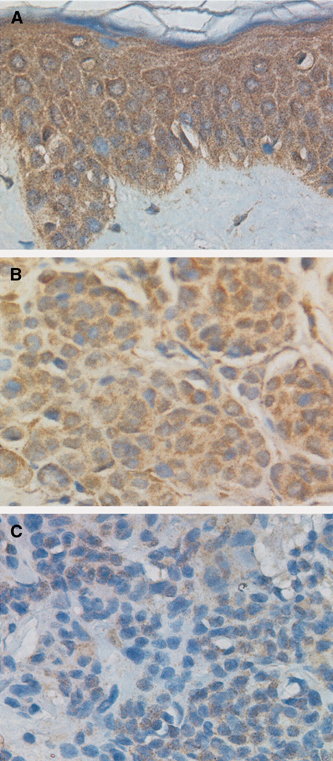
Apaf-1 expression in human melanoma tissue microarray. (**A**) Adjacent normal epidermis with strong Apaf-1 expression (3+). (**B**) Normal nevus with moderate Apaf-1 expression (2+). (**C**) Melanoma with weak Apaf-1 expression (1+). Magnification, × 400.

**Figure 2:**
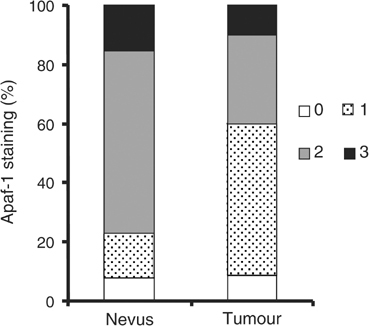
Apaf-1 expression in normal nevi and melanoma. There is significantly less Apaf-1 expression in tumour tissues compared to normal nevi (*P*=0.014, *χ*^2^ test).

We examined Apaf-1 expression in primary melanomas and nevi by immunohistochemistry. Various levels of Apaf-1 expression were observed in the cytoplasm of the biopsies (Figure 1

). Among the 70 melanoma primaries, six cases (8.6%) showed negative (0), 36 cases (51.4%) weak (1+), 21 cases (30%) moderate (2+), and seven cases (10%) strong (3+) Apaf-1 staining. In contrast to melanoma primaries, majority (76.9%) of the nevi had moderate or strong Apaf-1 expression (eight and two cases, respectively), while only one case stained negative and two cases showed weak Apaf-1 staining (Figure 2

). A significant difference in the staining pattern between tumours and nevi was observed (*χ*^2^=6.02, *P*=0.014).

### Apaf-1 expression and clinicopathological parameters or 5-year survival

**Figure 3:**
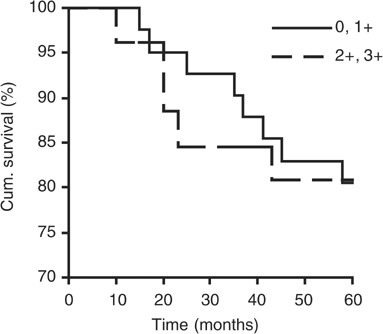
Correlation between Apaf-1 expression and 5-year patient survival. Apaf-1 expression is not correlated to 5-year patient survival (*P*>0.05, log-rank test).

To assess whether reduced Apaf-1 expression is associated with melanoma progression, we examined Apaf-1 expression in 70 melanoma primaries at various stages of invasion. As shown in Table 1, tumours with negative or weak Apaf-1 expression were distributed fairly evenly among the various Breslow thickness categories (*P*>0.05, *χ*^2^ test). Tumour ulceration is often considered an indicator for melanoma prognosis (Vihinen *et al*, 2003), but in our study, we did not find any correlation between Apaf-1 expression and tumour ulceration status (*P*>0.05, *χ*^2^ test). In addition, we did not find correlation between Apaf-1 expression with age, sex, tumour subtype, or location of tumours (sun protected *vs* sun exposed) (Table 1). To evaluate whether Apaf-1 staining might be related to patient survival, a Kaplin–Meier survival curve was constructed using overall 5-year survival to evaluate the biopsies stained negative or weak (0, 1+) *vs* those stained moderate or strong (2+, 3+) for Apaf-1 expression (Figure 3

). Our data did not show a correlation between Apaf-1 staining and 5-year patients survival (*P*>0.05, log-rank test).

### Overexpression of Apaf-1 enhances chemosensitivity in melanoma cells

**Figure 4:**
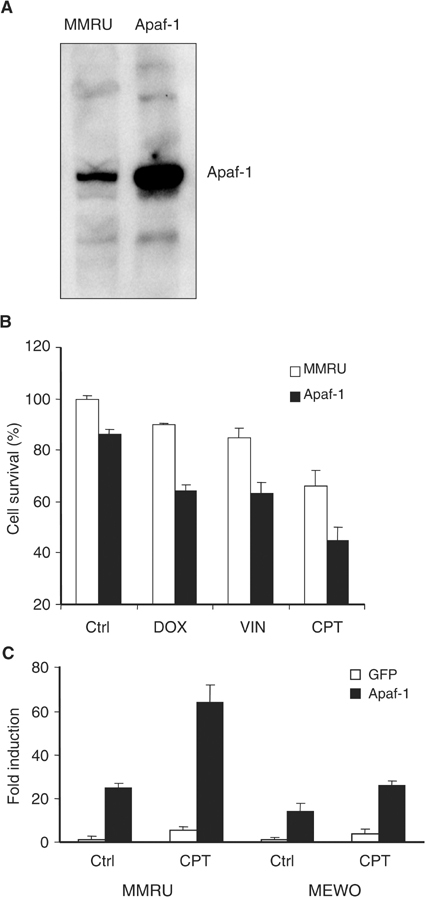
Overexpression of Apaf-1 sensitises melanoma cells to anticancer drug treatment. (**A**) Western blot showing endogenous level of Apaf-1 parental MMRU and MMRU cells transfected with Apaf-1. (**B**) Decreased cell survival of melanoma cells overexpressing Apaf-1. MMRU cells were either transfected with control GFP or Apaf-1 cDNAs followed by various drug treatment: DOX (500 nM), VIN (800 nM), or CPT (200 nM) for 24 h, and the cell survival rate was determined by SRB assay. (**C**) Increased apoptosis of melanoma cells overexpressing Apaf-1. MMRU and MEWO cells were transfected with GFP or Apaf-1, and then treated with 200 nM CPT for 24 h followed by ELISA assay of apoptosis.

To investigate if Apaf-1 can increase the chemosensitivity in melanoma cells, MMRU cells were overexpressed with Apaf-1 by transient transfection (Figure 4A

), followed by various anticancer drug treatments. Cell survival assay revealed that overexpression of Apaf-1 reduced cell survival to 63, 62 and 43% after treatment with doxorubicin, vincristine or camptothecin, respectively, compared with drug treatment alone (88, 83 and 66%) (Figure 4B). Our data showed that camptothecin (CPT) was the most effective drug at reducing cell survival with the concentrations used for the experiment. To further determine the extent of apoptosis, we then used an ELISA-based protocol, which quantifies the number of histone/DNA particles that are cleaved during the apoptotic process. As shown in Figure 4C, overexpression of Apaf-1 in MMRU cells increased apoptosis by 25-fold over the GFP-transfected control cells while this value was more than doubled (64-fold) after 200 nM CPT treatment. Furthermore, in a mutant-p53 MEWO cell line, overexpression of Apaf-1 induced 14- and 26-fold apoptosis in control and 200 nM CPT-treated cells, respectively (Figure 4C). These results indicate that Apaf-1 can sensitise melanoma cells to chemotherapy.

## DISCUSSION

Acquired resistance to apoptosis is a hallmark of cancers (Hanahan and Weinberg, 2000), which can allow cancer cells to survive and enable the establishment of metastasis or resistance to chemotherapy. Malignant melanoma is a particularly aggressive form of cancer in this regard. It is both highly metastatic and resistant to chemotherapy. Many chemotherapeutic agents, such as taxanes, vinca alkaloids and platinum-associated drugs, have failed to introduce a significant response in melanomas. Even DTIC, the only FDA-approved drug for the treatment of malignant melanoma can only cure 5–10% of patients (Soengas and Lowe, 2003).

Defects in the apoptotic pathways can be the cause of tumour progression and resistance to chemotherapy. Inactivation of Apaf-1, a key effector of the intrinsic apoptosis pathway, and its effect on chemotherapy have been observed in several tumour types, including gastrointestinal cancer, leukemia, ovarian cancer and melanoma (Yamamoto *et al*, 2000; Jia *et al*, 2001; Soengas *et al*, 2001; Wolf *et al*, 2001; Liu *et al*, 2002). In this study, to better understand the role of Apaf-1 in melanoma development, we used TMA technology and immunohistochemistry to investigate Apaf-1 expression level in primary human melanoma biopsies. Our results demonstrated that Apaf-1 expression is significantly reduced in melanoma compared to normal nevi (*P*=0.014) (Figure 2). However, despite the Apaf-1 reduction in melanoma, our data showed that Apaf-1 expression is not related to melanoma thickness (Table 1) or 5-year patient survival (Figure 3), suggesting that Apaf-1 reduction is an early event of melanoma tumorigenesis, possibly at the initiation stage.

The reduction of Apaf-1 expression in melanoma biopsies observed in our study is in agreement with previous studies demonstrating inactivation of Apaf-1 in melanoma cell lines (Soengas *et al*, 2001). The reversion of Apaf-1 expression by the methylation inhibitor 5-aza-2′-deoxycytidine in melanoma cells (Soengas *et al*, 2001) suggests that hypermethylation of Apaf-1 promoter or the upstream regulators may contribute to the reduction/loss of Apaf-1 expression in melanoma. Normal expression of Apaf-1 is an important component in the p53/mitochondrial intrinsic apoptosis pathway. Proapoptotic signal from p53 can result in the release of cytochrome *c* from the mitochondria. In the presence of cytochrome *c* and dATP, Apaf-1 binds to procaspase-9, leading to caspase-9 activation and initiation of a protease cascade (Li *et al*, 1997). Thus, reduced Apaf-1 expression in melanoma may terminate the apoptotic signal from mitochondria, thus disabling the p53 apoptotic program.

Our data that Apaf-1 reduction did not correlate with tumour thickness is in agreement with the findings by Fujimoto *et al* (2004), who showed that the pattern of *Apaf-1* LOH did not correlate with tumour Breslow thickness, while Apaf-1 mRNA expression level was significantly lower in Apaf-1 LOH positive tumours. Contrarily, Baldi *et al* (2004) reported that reduced Apaf-1 expression correlated with melanoma thickness. The following reasons may result in the discrepancy between our results and those by Baldi *et al*: (1) different antibodies used for immunohistochemical staining; and (2) different technical approaches for immunohistochemisty. We used TMA technique for our study and all the nevi and tumour biopsies were assembled in one slide. Therefore, all the samples had exactly the same treatment for each step during immunohistochemical staining. On the other hand, Baldi *et al* used regular immunohistochemistry for their study, so there may exist inconsistency between different slides during the staining procedure for 106 biopsies. Future TMA studies of additional tumour biopsies will validate the role of Apaf-1 in melanoma progression.

Our finding that no significant correlation between reduced Apaf-1 expression and tumour thickness favours a multiple-event model that a number of important molecular changes occur sequentially during melanoma progression. Dysfunction of other components in the apoptotic pathway as well as the survival pathway may contribute to melanoma progression. For example, survivin, a member of the inhibitor of apoptosis protein (IAP) family, was strongly expressed in human melanomas but not in normal melanocytes, and overexpression of survivin in the sentinel lymph nodes from melanoma patients was inversely correlated with patient survival (Grossman *et al*, 1999; Gradilone *et al*, 2003). High expression of Bcl-2 antiapoptotic proteins, such as Bcl-2, Bcl-X_L_, and Mcl-1, may also contribute to melanoma progression and chemoresistance as antisense oligos against these genes can induce death of melanoma cells (Jansen *et al*, 1998; Heere-Ress *et al*, 2002; Thallinger *et al*, 2003). However, the implication of Bcl-2 antiapoptotic proteins as melanoma progression factors is controversial. While some studies indicated that Bcl-2 and Bcl-X_L_ gene expression increases with progression of malignant melanoma (Leiter *et al*, 2000; Utikal *et al*, 2002), others found that Bcl-2 and Bcl-X_L_ did not correlate to progression of the disease (Gradilone *et al*, 2003). In the PI3K/AKT/PTEN survival pathway, AKT has been found to be constitutively activated in melanoma, which leads to upregulation of NF*κ*B and tumour progression (Dhawan *et al*, 2002). Recently, we demonstrated that the integrin linked kinase (ILK), a direct regulator of AKT activity, is overexpressed in melanoma and increased ILK expression is correlated with melanoma thickness and 5-year patient survival (Dai *et al*, 2003). As the negative regulator of this pathway, PTEN expression was found to be reduced in melanoma biopsies and loss of PTEN can promote tumour growth *in vivo* (Stahl *et al*, 2003; Tsao *et al*, 2003). Based on the complexity of the apoptotic and survival pathways that control the fate of a cell, additional studies on the timing of the gene inactivation/overexpression in these pathways from the same set of tumour biopsies and the interdependence among these events will provide a more complete picture of the molecular changes during melanoma initiation and progression. Given the fact that many factors participate in the governance of the fate of melanoma cells, it is worth targeting multiple molecules in different pathways as a therapeutic approach.

Our data that reduced Apaf-1 expression did not correlate with 5-year survival of patients with primary melanoma is consistent with the findings by Fujimoto *et al* (2004), who showed that Apaf-1 LOH correlated with poorer prognosis of metastatic, but not primary melanoma patients. These data also suggest that the involvement of Apaf-1 in melanoma tumorigenesis is very complex. Loss of Apaf-1 may trigger the initiation of malignant transformation of melanocytes. However, additional genetic changes are required for the vertical growth phase progression of melanoma. Nevertheless, overexpression of Apaf-1 in melanoma cells sensitised melanoma cells to anticancer drug treatment (Figure 4), suggesting that loss of Apaf-1 expression may cause chemoresistance in melanoma. Since the Apaf-1 expression is significantly reduced in melanomas and overexpression of Apaf-1 enhances anticancer drug-induced apoptosis, reversion of the reduced Apaf-1 expression should be considered in the design of novel strategies for the treatment of melanoma patients.
